# Three-dimensional exoscope-assisted laser stapedotomy: a preliminary experience

**DOI:** 10.1007/s00405-021-06672-1

**Published:** 2021-02-17

**Authors:** Umberto Milanesi, Benedetta Pasquariello, Alberto Maria Saibene, Giovanni Felisati, Murat Atac, Davide Corbetta

**Affiliations:** 1 Otolaryngology Unit, Desio Hospital-ASST Brianza, Desio, Italy; 2 Otolaryngology Unit, Department of Health Sciences, ASST Santi Paolo e Carlo, Università degli Studi di Milano, Milan, Italy

**Keywords:** Three-dimensional vision, Exoscope, Stapedotomy, Otosclerosis, Video-assisted surgery

## Abstract

**Purpose:**

Stapes surgery, despite the introduction of lasers and endoscopes, still represents a challenging procedure. Recently introduced 3-dimensional exoscopes have known advantages in otological surgery. This study aims to evaluate exoscopes role in stapes surgery, both from a surgical perspective and on the educational profile.

**Methods:**

Seven consecutive otosclerosis patients underwent single-sided endaural laser stapedotomy with a 4K 3-dimensional exoscope. The surgical setting allowed all operating room personnel 3-dimensional vision. Pre- and postoperative pure tone audiometry and air-bone gaps, and information on the postoperative course and complications were systematically collected. An informal ergonomic evaluation was carried out by the operating room personnel and an informal didactic evaluation was provided by the trainees. A comparable group of microscope-assisted stapedotomy patients undergoing the same procedures and evaluations was chosen as a control group.

**Results:**

Outcomes were solid in all patients, median air-bone gap decreased from 26.5 to 10 dB at the 3-month evaluation (*p* = 0.01, Wilcoxon’s test). No vertigo, tinnitus, or facial palsy was reported. The median operating time was 40 min. The compact design and configuration of the exoscope allowed more practical management of the operating theater. All personnel had the chance for a better understanding of the procedure and trainees felt more confident when asked to identify surgical landmarks and procedure steps. Audiological outcomes, operative times, and complication rates were not different between study and control groups.

**Conclusion:**

Though further validation and systematic comparison with microscope- and endoscope-assisted stapedotomy are required, the exoscope proved a safe, practical, and educational tool.

**Supplementary Information:**

The online version contains supplementary material available at 10.1007/s00405-021-06672-1.

## Introduction

Despite a significant standardization, stapes surgery remains a technically challenging procedure with little error margin [1]. Results depend on the correct indication, proper middle ear exposure, reproducible surgical sequences, surgical dexterity, and good visualization of the critical areas during the whole procedure [2]. The endaural approach according to Fisch is a traditional technique that stood the test of time [3]. In the last 20 years, stapes surgery saw two major innovations: the introduction of different lasers in place of micro drills [4] and the development of exclusively endoscopic techniques replacing the standard use of microscopes [5]. Exoscopes, most often equipped with high-definition and 3-dimensional (3D) sensors, are the newest addition in terms of surgical field visualization and proved a solid tool in the fields of neurosurgery, neuro-otological surgery, and head and neck oncological surgery [6–10]. The 3D digital image system allows the surgeon, wearing special glasses, to perform the procedure using a magnified image of the field on a 4K 3D monitor with significant comfort also for assistants and nurses, who are also granted magnified 3D vision. Though other otological applications have been already studied in selected case series [11], no study preliminarily evaluated the role of exoscope-assisted surgery in otosclerosis. This study aims to investigate the advantages, ergonomics, didactic value of a 4K 3D exoscopic system in performing endaural laser-assisted stapedotomy in a prospective series of stapedotomy patients, compared with a control group of microscope-assisted stapedotomy patients.

## Material and methods

All procedures described in this report were performed in accordance with the declaration of Helsinki and we received informed consent for the procedures from all participating patients.

### Patients

We prospectively included in this series only patients with a clinical diagnosis of otosclerosis (single- or double-sided) with an air-bone gap (ABG) in the affected ear(s) of at least 35 dB (pure tone average, PTA, 500–1000–2000–4000 Hz).

Exclusion criteria were: anesthesiological contraindications to general anesthesia, prior otological surgery, monaural hearing, prior cochlear implantation, pediatric age.

Age and sex were the only demographics variables recorded for the study.

### Pre- and post-surgical evaluation

All patients underwent the following evaluations: pure tone audiometry with air and bone thresholds (pre-operative and 3-months post-operative) and facial movements evaluation (pre-operative, immediate post-operative, and 3-months postoperative). Furthermore, the patients were specifically asked for symptoms of tinnitus or vertigo pre-, and post-operatively (both immediately and 3-months after the procedure).

### Surgical procedure

All eligible patients underwent single-sided laser stapedotomy under general anesthesia. In the case of bilateral otosclerosis, the procedure was planned in the worse-hearing ear.

The procedure followed the “one-shot” laser stapedotomy technique detailed by Poletti et al. [12].

The surgical instrumentation was as follows:

1. Fisch stapedotomy-dedicated otological surgery instrumentation (Karl Storz, Tuttlingen, Germany)
2. Surgical 3D exoscope system, composed as follows (all components are manufactured by Karl Storz, Tuttlingen, Germany): 3D exoscope (VITOM 3D), holding arm (Versacrane), joystick for exoscope and zoom control (Image1 Pilot), light source (Power Led 300), camera controller (Image1 S Connect). The system was connected to a 32-inches 3D monitor. Surgery was performed by watching the video transmission on the monitor and wearing 3D polarization glasses.
3. 940 nm diode laser (Medilas D, Dornier MedTech, Wessling, Germany) delivered through a 0.6 mm fiber. The laser setting was high power, 40 W, set with a short 60-ms single pulse mode
4. Fisch titanium stapes piston 8.5 × 0.4 mm (Karl Storz, Tuttlingen, Germany). The prostheses were further cut according to the distance between the footplate and the lateral surface of the incus, after measurement with the specific malleable tool. This distance was further increased by 0.5 mm taking into consideration the protrusion of the titanium piston into the vestibule. The prostheses were trimmed on the special cutting block.

**Fig. 1:**
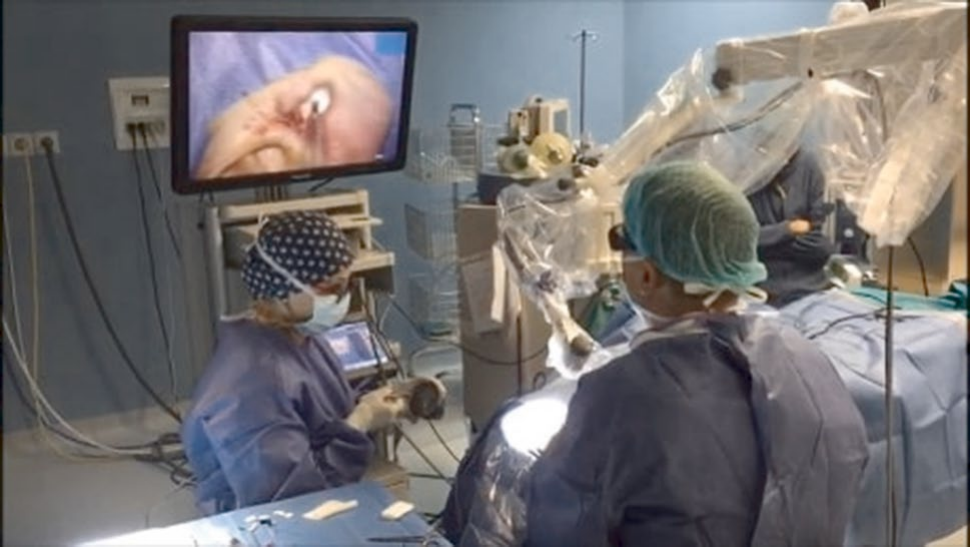
The image shows the operating theatre setting, with the scrub nurse positioned at the head of the patient and the surgeon in the head-up position while operating the right side ear

**Fig. 2:**
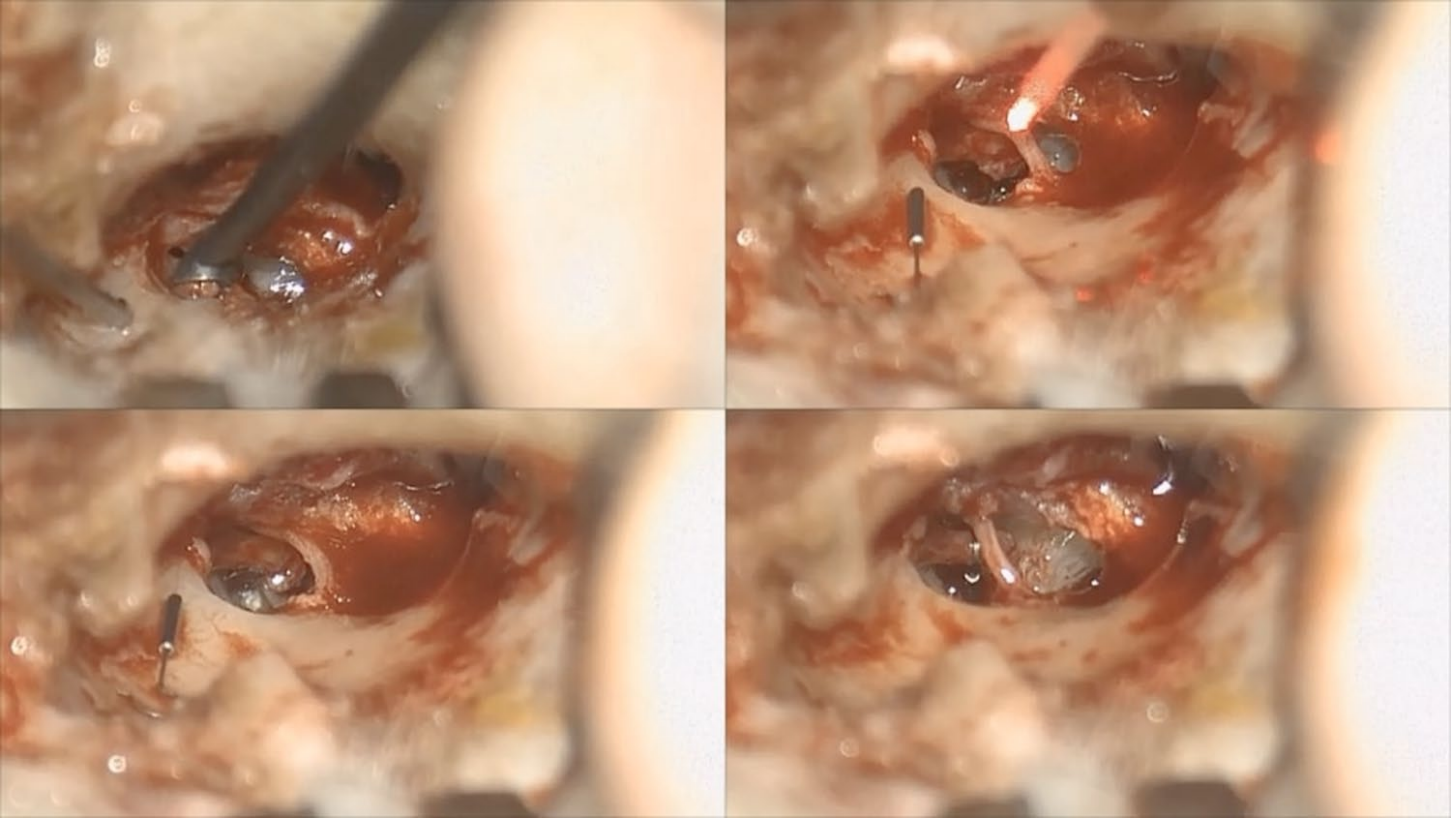
The image shows different steps of the surgical procedure viewed through the exoscope. Upper left: curettage of the posterosuperior bony ear canal. Lower left: oval window exposure with the titanium piston visible in the surgical field. Upper right: one-shot diode laser stapedotomy. Lower right: titanium piston crimped to the long process of the incus

The procedure was entirely performed with the aid of the exoscopic system by a single senior surgeon (UM). The operating theater setting with the surgeon and the scrub nurse is shown in Fig. 1. The camera was positioned below and anterior to the surgeon, about 30 cm from the surgical field. The endaural approach allowed a wide surgical field exposition, sufficient for the exoscopic vision. Linder and Fisch’s checklist for stapes surgery [13] was followed to ensure proper endaural vision. The standardized surgical steps were: endaural incision, positioning of two orthogonally positioned retractors, wide tympanomeatal flap preparation, scutum curettage, chorda tympani mobilization, ossicular chain motility testing (aimed at confirming stapes fixity), stapedial tendon and posterior crus diode laser section, footplate-lateral incus surface distance measurement, preparation of the titanium prosthesis on the block, piston locking in the special holder, one-shot laser stapedotomy, prosthesis placement, loop tightening to the long process of the incus (Fig. 2), anterior crus section and down fracture, stapedial superstructure removal, and prosthesis and connected ossicular chain correct motility checking. After final checks, the tympanomeatal flap was repositioned and held in place with a 4/0 polyglactin absorbable braided suture and dressing with porcine gelatine in the external auditory canal.

### Ergonomic and didactic value evaluation

The senior surgeon, 2 different assistants, and 2 scrub nurses were requested to report their personal ergonomic evaluation compared with standard microscope-assisted stapedotomy.

2 different trainees were instructed to identify the anatomic landmarks during the procedure and report on the didactic value of the surgical session.

### Comparison with microscope-surgery assisted stapedotomy

A randomly chosen sample of 7 consecutive patients undergoing endoscope-assisted laser stapedotomy with the same inclusion and exclusion criteria as the study group was used as a control group. The control group underwent the exact same protocol as the study group, the only exception being the use of a standard binocular operating microscope (OPMI Sensera 7, Carl Zeiss Meditec AG, Jena, Germany). Baseline characteristics and results were compared between the two groups.

### Data collection and statistical analysis

All data collected for this study were pseudonymized and collected into a Google Sheets spreadsheet (Google LLC, Mountain View, CA, US). We performed descriptive statistics and statistical analysis of the collected data to compare pre- and postoperative auditory thresholds. The sample size was considered too small to assume a normal distribution. Pre- and post-operative comparisons in the study group were performed with Wilcoxon’s signed-rank test. Inter-group comparisons in terms of audiological characteristics and results, and operative time were performed with a Mann–Whitney *U* test and binary variables (sex, complications rates) were performed with a Fisher’s test. All statistical analyses were performed using the SPSS software, Version 25.0. (IBM Corporation, Armonk, NY, US). Tests were two-tailed and values of *p* < 0.05 were considered to indicate statistical significance. Unless otherwise stated, for descriptive statistics data are reported as median ± interquartile range (minimum value–maximum value).

## Results

In the study period between September 2019 and February 2020, 7 consecutive patients (4 male and 3 female) meeting the inclusion criteria were identified. All patients were included in the study after careful evaluation of the inclusion and exclusion criteria. Age was 48 ± 22.5 (29–67) years. Preoperative air conduction PTA was 56.25 ± 16.25 (25–76.25) dB. The preoperative PTA air-bone gap was 26.25 ± 21.75 (16.25–41.25) dB. Three-months postoperative air conduction PTA was 36.25 ± 13.13 (23.75–47.5) dB. Three-months postoperative PTA air-bone gap was 10 ± 3.5 (8.75–27.5) dB. The variation was statistically significant both in air conduction PTA and PTA air-bone gap (Wilcoxon’s signed-rank test, *p* = 0.038, and *p* = 0.01, respectively). Preoperative bone conduction PTA was 26.25 ± 13.75 (11.25–46.25) dB, while postoperative bone conduction PTA was 17.5 ± 12 (15–38.75) dB. This variation wasn’t statistically significant (Wilcoxon’s signed-rank test, *p* = 0.749). Full demographic and audiometric data for each patient undergoing exoscope-assisted stapedotomy are shown in Table 1.

**Table 1:** Demographic and audiometric data for patients undergoing exoscope-assisted stapedotomy

Patient ID	Sex	Age (years)	Pure tone audiometry (500–1000–2000–4000 Hz)						4 kHz bone conduction preoperative/3-months postoperative variation	8 kHz air conduction preoperative/3-months postoperative variation
			Air conduction		Bone conduction		Air–bone gap			
			Preoperative	3-months postoperative	Preoperative	3-months postoperative	Preoperative	3-months postoperative		
GA	M	48	76.25	45	26.25	17.5	50	27.5	0	15
PM	F	56	53.75	47.5	37.5	38.75	16.5	8.75	− 10	10
TP	M	67	43.75	36.25	27.5	26.25	16.25	10	− 5	− 5
BLC	F	64	72.5	42.5	46.25	32.5	26.25	10	0	10
FB	F	38	56.25	23.75	21.25	15	35	8.75	0	30
OJ	M	29	57.5	32.5	16.25	17.25	41.25	15.25	0	5
SN	M	37	25	28.75	11.25	17.5	15.75	10.5	− 15	10

None of the patients had objective preoperative VII cranial nerve palsy or reported subjective tinnitus or vertigo. These three parameters were unchanged both on postoperative day 1 and at the 3-month evaluation. There were no intraoperative or postoperative complications. All patients were dismissed on a postoperative day 1.

The surgical procedure duration in the study group was 35 ± 5 (35–45) min. The preparation and positioning of the exoscopic system were practical and required no modification during the procedure. Anatomic landmarks were easily recognized by surgeons and trainees with a single position of the exoscope articulated arm. The senior surgeon position was more comfortable than in microscope-assisted procedures, given the horizontal and direct line of sight to the monitor. The presence of 3D glasses did not disturb surgeons and scrub nurses despite having to look at both monitor and surgical instruments with a comparable feel with microscope-assisted procedures. The 3D vision allowed all the operative room staff a better understanding of the surgical steps and appreciation of precision movements, with a significant didactic value for trainees.

### Comparison with microscope-surgery assisted stapedotomy

The 7-patients control group showed similar baseline characteristics, operative data, and results as the study group. Full demographic and audiometric data for each patient undergoing microscope-assisted stapedotomy are shown in Online Resource 1 and statistical comparisons between groups are reported in Online Resource 2.

Operative time in the control group was 35 ± 7.5(35–50) minutes, therefore, not statistically different from the study group (*p* = 1, Mann–Whitney *U* test).

## Discussion

The 3D exoscopic technique has been successfully tested in various neurotological procedures by different authors [6, 7, 10, 14, 15] but, to the best of our knowledge, this is the first case described in the literature where this instrumentation is used for an endaural stapedotomy.

Our article shows that Fisch’s stapedotomy via endaural approach can be reproducibly and safely performed using a 3D exoscopic system. Furthermore, the 3D surgical field vision on a frontal monitor is particularly comfortable for the surgeon, while the whole setting is both ergonomic for the whole operating room personnel and extremely effective as a didactic tool for trainees. Preliminary surgical outcomes, operative times, and complication rates appear analogous to the traditional microscopic technique, as further demonstrated by the direct comparison between study and control groups.

Despite the small patients’ batch, our data suggest that 3D exoscopes might represent in the future significant contenders to microscopes in stapes surgery. The compact design and configuration of exoscopes definitely reduce the vision system bulk in the operating theater when compared to microscopes.

Exoscope advantages include high-resolution 3D visualization, increased degree of freedom for image adjustment, and reduced visual and postural surgeon fatigue [6, 7, 10, 14]. It is well-known that microscope-assisted surgery forces the surgeon in a reclined position in line with the eyepieces, increasing the risk of musculoskeletal fatigue and pain [16]. The head-up surgery position frontal to the monitor is much more ergonomic and comfortable [14]. Furthermore, while both microscope and exoscope remain outside the surgical field, the latter grants 3D vision to the whole surgical team, immersing them in the operator’s perspective [6, 8–11]. While surgical steps are the same for microscopy and exoscopy, without the need to reposition the articulated arm, the exoscope independent joystick can be controlled by the assistant for zoom, brightness, and image capture without intervention from the operator [8, 10]. It is interesting to note that we modified the operating room setting with regard to what Minoda and Miwa proposed [14]. The articulated arm and the exoscope are positioned right of the operator while the scrub nurse sits at the head of the patient. We believe that this position allows at the same time to assist the surgeon and follow the procedure on video.

Furthermore, we tested the effective possibility offered by the exoscope to correctly visualize the operating field according to the checklist proposed by Linder and Fisch [13]. The exoscope allows seeing all the four relevant anatomical landmarks in a single field no differently than with a standard microscope. It has to be noted that in our experience the endaural approach is mandatory, as transcanalar approaches do not seem to grant a sufficient visualization of the field with the exoscope.

The learning curve is almost imperceptible to surgeons already trained in using microsurgical instruments, as previously reported [6, 7, 10], whilst the possibilities offered in otological surgery training remain to be explored and appear appalling given the significant didactical feedback we received.

Our experience also confirms some already described limitations of the system, most specifically insufficient light conveying in small surgical corridors and the loss of signal-to-noise ratio at higher magnification levels [8, 10, 14]. The insufficient resolution to grant impeccable view at higher magnification could represent a major issue should the surgeon requires checking the correct positioning of the prosthesis or a suspected perilymphatic leakage around the piston, two possible scenarios that luckily we weren’t required to face. Nevertheless, it has to be taken into account that excellent results have been already described in restricted surgical fields (e.g. posterior tympanotomy for cochlear implantation [6]).

A major present limitation of exoscope assisted surgery is represented by its cost. The full exoscopic system used for this article has a price market of roughly 120,000 € (with more than 40% of the cost accounting to image processing and display units for 3D vision) while the microscope we used for comparison in this work costs about 47,000 € (although this latter price does not comprise display units, which are indeed not strictly required for standard microscope-assisted stapedotomy). This excess cost in our opinion could be adequately offset over about 100 procedures per year. This makes exoscopes recommendable at present only for high or very high-volume teaching hospitals, where the higher cost is counterbalanced by the educational value. Furthermore, most otolaryngological facilities are currently already equipped with an operating microscope and the personnel is already trained in its use, so exoscope remains at present a further tool and not a replacement, with a significant overall healthcare expenditure required. Nevertheless, more complex cost-effectiveness analyses, taking into account surgical, clinical, ergonomic, and teaching aspects over the whole range of procedures open to exoscopic assistance would be required.

Albeit our results have been collected prospectively and provide an agile comparison with microscope-assisted surgery, our study lacks a significant bulk of patients, and only future randomized trials and more focused comparison might help define and circumstance the peculiarities of exoscopy in stapes surgery. Up to then, this visual instrumentation remains another useful tool in otological surgery with relevant didactical implications.

## Supplementary Information

Below is the link to the electronic supplementary material.

Supplementary file1 (PDF 228 KB)

Supplementary file2 (PDF 333 KB)

## Abbreviations

PTA: Pure tone audiometry
ABG: Air–bone gap

## References

[1] Fang L, Lin H, Zhang T-Y, Tan J (2014). Laser versus non-laser stapedotomy in otosclerosis: a systematic review and meta-analysis. Auris Nasus Larynx.

[2] Dhooge I, Desmedt S, Maly T, Loose D, Van Hoecke H (2018). Long-term hearing results of stapedotomy: analysis of factors affecting outcome. Eur Arch Otorhinolaryngol.

[3] Stapedotomy FU, Stapedectomy V (2009). Stapedotomy versus stapedectomy. Otol Neurotol.

[4] Kamalski DMA, Wegner I, Tange RA, Vincent R, Stegeman I, van der Heijden GJM (2014). Outcomes of different laser types in laser-assisted stapedotomy: a systematic review. Otol Neurotol.

[5] Nikolaos T, Aikaterini T, Dimitrios D, Sarantis B, John G, Eleana T (2018). Does endoscopic stapedotomy increase hearing restoration rates comparing to microscopic? A systematic review and meta-analysis. Eur Arch Otorhinolaryngol.

[6] Smith S, Kozin ED, Kanumuri VV, Barber SR, Backous D, Flávio Nogueira J (2019). Initial experience with 3-dimensional exoscope-assisted transmastoid and lateral skull base surgery. Otolaryngol Head Neck Surg.

[7] Garneau JC, Laitman BM, Cosetti MK, Hadjipanayis C, Wanna G (2019). The use of the exoscope in lateral skull base surgery: advantages and limitations. Otol Neurotol.

[8] Crosetti E, Arrigoni G, Manca A, Fantini M, Caracciolo A, Sardanapoli F (2020). VITOM-3D assisted neck dissection via a retroauricular approach (RAND-3D): a preclinical investigation in a cadaver lab. Acta Otorhinolaryngol Ital.

[9] De Virgilio A, Mercante G, Gaino F, Yiu P, Mondello T, Malvezzi L (2020). Preliminary clinical experience with the 4K3-dimensional microvideoscope (VITOM 3D) system for free flap head and neck reconstruction. Head Neck.

[10] Rubini A, Di Gioia S, Marchioni D (2020). 3D exoscopic surgery of lateral skull base. Eur Arch Otorhinolaryngol.

[11] Colombo G, Ferreli F, Di Bari M, Cugini G, Miceli S, De Virgilio A (2021). Introducing the high-definition 3D exoscope in ear surgery: preliminary analysis of advantages and limits compared with operative microscope. Eur Arch Otorhinolaryngol.

[12] Poletti AM, Miceli S, Rossi V, Di Pietro S, Tosi G, Colombo G (2015). The “One Shot” diode laser stapedotomy. Photomed Laser Surg.

[13] Linder TE, Fisch U (2007). A checklist for surgical exposure in stapes surgery: how to avoid misapprehension. Adv Otorhinolaryngol.

[14] Minoda R, Miwa T (2019). Non-microscopic middle ear cholesteatoma surgery: a case report of a novel head-up approach. Otol Neurotol.

[15] Garneau JC, Laitman BM, Cosetti MK, Hadjipanayis C, Wanna GB (2020). Repair of a temporal bone encephalocele with the surgical exoscope. Otol Neurotol.

[16] Cohen-Gadol AA (2020). Surgeon’s philosophy and ergonomic operating position: advancing efficiency and minimizing fatigue during microsurgery. World Neurosurg.

