# Supplementary material for: Three-dimensional exoscope-assisted laser stapedotomy: a preliminary experience
Source: Eur Arch Otorhinolaryngol. 2021 Feb 17;278(11):4593–8. doi: 10.1007/s00405-021-06672-1 (PMC8486714; doi:10.1007/s00405-021-06672-1)
Supplement: Supplementary file 1 — Supplementary file1 (PDF 228 KB) [file 405_2021_6672_MOESM1_ESM.pdf]

THREE-DIMENSIONAL EXOSCOPE-ASSISTED LASER STAPEDOTOMY: A PRELIMINARY EXPERIENCE

Journal: European Archives of Oto-Rhino-Laryngology

Authors: Milanesi Umberto, Pasquariello Benedetta, Saibene Alberto Maria, Felisati Giovanni, Atac Murat, Corbetta Davide

Correspondence to: Alberto Maria Saibene, Otolaryngology Unit - ASST Santi Paolo e Carlo. Via Antonio di Rudinì, 8 - 20142 - Milan, Italy. Phone: +39 02 8184 4249. Fax: +39 02 5032 3166. Mail: alberto.saibene@unimi.it

Online resource 1: Demographic and audiometric data for patients undergoing microscope-assisted stapedotomy

Patient ID	Sex	Age (years)	Pure Tone Audiometry (500-1000-2000-4000 Hz)				Air-bone gap		4 kHz bone conduction preoperative/ 3-months postoperative variation	8 kHz air conduction preoperative/ 3-months postoperative variation
			Air conduction		Bone conduction					
			Preoperative	3-months postoperative	Preoperative	3-months postoperative	Preoperative	3-months postoperative		
BC	M	38	53.75	40	15	15	38.75	25	0	-5
CA	F	51	76.25	33.75	41.25	26.25	35	7.5	5	15
LL	M	48	47.5	23.75	20	13.75	27.5	10	0	10
LS	F	41	46.25	42.5	17.5	21.25	28.75	21.25	-20	-45
BE	F	56	74	67	37.5	38.75	36.5	28.25	-5	-5
MA	F	36	59	31	26.25	20	32.75	11	0	20
LR	M	42	25	28.75	11.25	17.5	15.75	10.5	-15	10
