# Supplementary material for: Three-dimensional exoscope-assisted laser stapedotomy: a preliminary experience
Source: Eur Arch Otorhinolaryngol. 2021 Feb 17;278(11):4593–8. doi: 10.1007/s00405-021-06672-1 (PMC8486714; doi:10.1007/s00405-021-06672-1)
Supplement: Supplementary file 2 — Supplementary file2 (PDF 333 KB) [file 405_2021_6672_MOESM2_ESM.pdf]

THREE-DIMENSIONAL EXOSCOPE-ASSISTED LASER STAPEDOTOMY: A PRELIMINARY EXPERIENCE

Journal: European Archives of Oto-Rhino-Laryngology

Authors: Milanesi Umberto, Pasquariello Benedetta, Saibene Alberto Maria, Felisati Giovanni, Atac Murat, Corbetta Davide

Correspondence to: Alberto Maria Saibene, Otolaryngology Unit - ASST Santi Paolo e Carlo. Via Antonio di Rudinì, 8 - 20142 - Milan, Italy. Phone: +39 02 8184 4249. Fax: +39 02 5032 3166. Mail: alberto.saibene@unimi.it

Online resource 2: Comparison of demographics, operative times, pre- and post-operative audiological data and complication rates between study and control groups.

<table>
<tr><th colspan="3"></th><th>Study Group (Exoscope- assisted)</th><th>Control Group (Microscope-assisted)</th><th>Statistical test used for comparison and p value, where available</th></tr>
<tr><td colspan="3">Age at surgery (years)</td><td>48 ± 22.5 (29-67)</td><td>42±10(36-56)</td><td>p= .749, Mann-Whitney U test</td></tr>
<tr><td colspan="3">Sex distribution (F:M) (N)</td><td>3:4</td><td>4:3</td><td>p= 1, Fisher's test</td></tr>
<tr><td colspan="3">Surgical time (minutes)</td><td>35±5(35-45)</td><td>35±7.5(35-50)</td><td>p= 1, Mann-Whitney U test</td></tr>
<tr><td colspan="3">Preoperative VII cranial nerve deficit rate (yes:no)</td><td>0:7</td><td>0:7</td><td>p= 1, Fisher's test</td></tr>
<tr><td colspan="3">Postoperative VII cranial nerve deficit rate (yes:no)</td><td>0:7</td><td>0:7</td><td>p= 1, Fisher's test</td></tr>
<tr><td colspan="3">Preoperative tinnitus rate (yes:no) (N)</td><td>0:7</td><td>0:7</td><td>p= 1, Fisher's test</td></tr>
<tr><td colspan="3">Postoperative tinnitus rate (yes:no) (N)</td><td>0:7</td><td>0:7</td><td>p= 1, Fisher's test</td></tr>
<tr><td colspan="3">Preoperative vertigo rate (yes:no) (N)</td><td>0:7</td><td>0:7</td><td>p= 1, Fisher's test</td></tr>
<tr><td colspan="3">Postoperative vertigo rate rate (yes:no) (N)</td><td>0:7</td><td>0:7</td><td>p= 1, Fisher's test</td></tr>
<tr><td rowspan="6">Pure Tone Audiometry (500-1000-2000-4000 Hz) (dB)</td><td rowspan="2">Air conduction</td><td>Preoperative</td><td>56.25 ± 16.25 (25 - 76.25)</td><td>55±15.88 (46.25-76.25)</td><td>p= .795, Mann-Whitney U test</td></tr>
<tr><td>3-months postoperative</td><td>36.25 ± 13.13 (23.75-47.5)</td><td>40±11.88 (23.75-67)</td><td>p= .795, Mann-Whitney U test</td></tr>
<tr><td rowspan="2">Bone conduction</td><td>Preoperative</td><td>26.25±13.75(11.25-46.25)</td><td>26.25±15 (15-41.25)</td><td>p= 1, Mann-Whitney U test</td></tr>
<tr><td>3-months postoperative</td><td>17.5±12(15-38.75)</td><td>21.25±11.88 (13.75-38.75)</td><td>p= 1, Mann-Whitney U test</td></tr>
<tr><td rowspan="2">Air-bone gap</td><td>Preoperative</td><td>26.25±21.75 (16.25-41.25)</td><td>32.75±7.63 (25-38.75)</td><td>p= .57, Mann-Whitney U test</td></tr>
<tr><td>3-months postoperative</td><td>10±3.5 (8.75-27.5)</td><td>13.5±12.63 (7.5-28.25)</td><td>p= .37, Mann-Whitney U test</td></tr>
<tr><td colspan="3">4 kHz bone conduction preoperative/3-months postoperative variation (dB)</td><td>0±7.5 (-15-0)</td><td>0±5 (-20-13.5)</td><td>p= .41, Mann-Whitney U test</td></tr>
<tr><td colspan="3">8 kHz air conduction preoperative/3-months postoperative variation (dB)</td><td>10±5 (-5-30)</td><td>12.5±22.5 (-45-20)</td><td>p= .31, Mann-Whitney U test</td></tr>
</table>
